# A Wearable Low-Power Sensing Platform for Environmental and Health Monitoring: The Convergence Project

**DOI:** 10.3390/s21051802

**Published:** 2021-03-05

**Authors:** Elise Saoutieff, Tiziana Polichetti, Laurent Jouanet, Adrien Faucon, Audrey Vidal, Alexandre Pereira, Sébastien Boisseau, Thomas Ernst, Maria Lucia Miglietta, Brigida Alfano, Ettore Massera, Saverio De Vito, Do Hanh Ngan Bui, Philippe Benech, Tan-Phu Vuong, Carmen Moldovan, Yann Danlee, Thomas Walewyns, Sylvain Petre, Denis Flandre, Armands Ancans, Modris Greitans, Adrian M. Ionescu

**Affiliations:** 1 Univ. Grenoble Alpes, CEA, LETI, F-38000 Grenoble, France; laurent.jouanet@cea.fr (L.J.); adrien.faucon@cea.fr (A.F.); audrey.vidal@cea.fr (A.V.); sebastien.boisseau@cea.fr (S.B.); thomas.ernst@cea.fr (T.E.); 2 ENEA CR-Portici, TERIN-FSD Department, P.le E. Fermi 1, 80055 Portici, Italy; tiziana.polichetti@enea.it (T.P.); mara.miglietta@enea.it (M.L.M.); brigida.alfano@enea.it (B.A.); ettore.massera@enea.it (E.M.); saverio.devito@enea.it (S.D.V.); 3 Univ. Grenoble Alpes, CEA, LITEN, F-38000 Grenoble, France; alexandre.pereira@cea.fr; 4 GINP, IMEP-LAHC, INP Grenoble—Minatec, 3 Parvis Louis Néel, CS 50257, F-38016 Grenoble, France; do-hanh-ngan.bui@grenoble-inp.fr (D.H.N.B.); philippe.benech@grenoble-inp.fr (P.B.); tan-phu.vuong@minatec.inpg.fr (T.-P.V.); 5 National Institute for R&D in Microtechnologies, 077190 Voluntari, Romania; carmen.moldovan@imt.ro; 6 ICTEAM, Université Catholique de Louvain (UCLouvain), 1348 Louvain-la-Neuve, Belgium; yann.danlee@uclouvain.be (Y.D.); thomas.walewyns@uclouvain.be (T.W.); sylvain.petre@uclouvain.be (S.P.); denis.flandre@uclouvain.be (D.F.); 7 Institute of Electronics and Computer Science, 1006 Riga, Latvia; armands.ancans@edi.lv (A.A.); modris_greitans@edi.lv (M.G.); 8 NanoLab, Ecole Polytechnique Federale de Lausanne (EPFL), 1015 Lausanne, Switzerland; adrian.ionescu@epfl.ch

**Keywords:** wearable electronics, low-power consumption, integration, environment monitoring, health monitoring, NO_x_ sensor, CO sensor, autonomous sensing platform, Internet of Things (IoT)

## Abstract

The low-power sensing platform proposed by the Convergence project is foreseen as a wireless, low-power and multifunctional wearable system empowered by energy-efficient technologies. This will allow meeting the strict demands of life-style and healthcare applications in terms of autonomy for quasi-continuous collection of data for early-detection strategies. The system is compatible with different kinds of sensors, able to monitor not only health indicators of individual person (physical activity, core body temperature and biomarkers) but also the environment with chemical composition of the ambient air (NO_x_, CO_x_, NH_x_ particles) returning meaningful information on his/her exposure to dangerous (safety) or pollutant agents. In this article, we introduce the specifications and the design of the low-power sensing platform and the different sensors developed in the project, with a particular focus on pollutant sensing capabilities and specifically on NO_2_ sensor based on graphene and CO sensor based on polyaniline ink.

## 1. Introduction

The importance and emergence of the Internet-of-Things and connected devices, led us to develop new efficient solutions for preventive healthcare, environment and life-style [1,2].

In that context, the development of wearable platforms embedding bio and environment sensors is of high importance to enable personalized advices and assistance for health and interactions with the environment. The latter, when degraded by the presence of anthropogenic and natural pollutants, is widely recognized as the most effective driver of severe health conditions. Mainly, this occurs through long-term exposure to pollutants that can go unnoticed until the development of the actual condition [3]. Smart wearable platforms, if equipped with an adequate sensor array, could contribute to monitor and record exposure indexes to pollutants contributing to build up a knowledge base, called exposome, with potential impact on disease prevention and personalized healthcare development.

The purpose of the Convergence project is to develop and demonstrate a low-power wearable platform dedicated to monitoring by exploiting the convergence of multi-parameter devices such as bio-, activity and environmental sensors. A multi-sensors platform was thus designed for the acquisition of not only individual physical condition parameters (physical activity, core body temperature, sweat and pH) but also the chemical composition of the surrounding ambient air (NO_x_, CO_x_, NH_x_ compounds).

These systems will certainly become a part of Internet-of-Things (IoT) devices and related services for a quality-of-life and/or for paradigm changes in the medical field. More recently, this concept merged into the so-called Internet-of-Everything (IoE), which has become a catch-all phrase to describe the addition of connectivity and intelligence to just about every device in order to give them special functions.

During the Convergence project, wearable physical and physiological low power sensors were developed like a multisensor IMU sensor device [4], a sensor for the detection of the heart’s electrical activity [5] and Si nanonet FET (NNFET) aptasensors for electrical detection of a specific protein such as thrombin [6]. Different wearable environmental sensors (gas and particles) were studied for NO_2_, NH_3_, CO_2_ and CO [7] detection. The platform was designed to support fast early data analysis algorithms and low power and secure transmission so as to enable continuous and localized feedback to users through appropriate machine learning algorithms (e.g., activity and fall detection and/or real time air pollutant exposure assessment) also developed during the project [4,8,9].

In this paper, we proposed a generic low-power wearable sensing platform. Architecture and specifications will be detailed. A focus on the development of NO_2_ and CO sensors will be explained in details.

## 2. Low-Power Wearable Sensing Platform: Architecture, Specifications and Design

A low power, wireless demonstration platform for wearable IoT flexible systems was designed and developed in view of our specific application for health and environment monitoring. The core activity for combining various contributions into unique proof of concept demonstrators is based on previous works [10,11,12,13].

The low-power sensing platform is a wearable low-power system and consists of an electronic board with:

1. Analog and digital sensors
2. Data acquisition and visualization in real-time with specific a App developed by CEA-LETI (Grenoble, France)
3. Radio Frequency (RF) Microcontroller Unit (MCU)
4. Bluetooth Low Energy (BLE) 2.4 GHz communication (data collection on mobile phone)
5. Antenna circuit designed by our G-INP partner (Grenoble, France).

**Figure 1:**
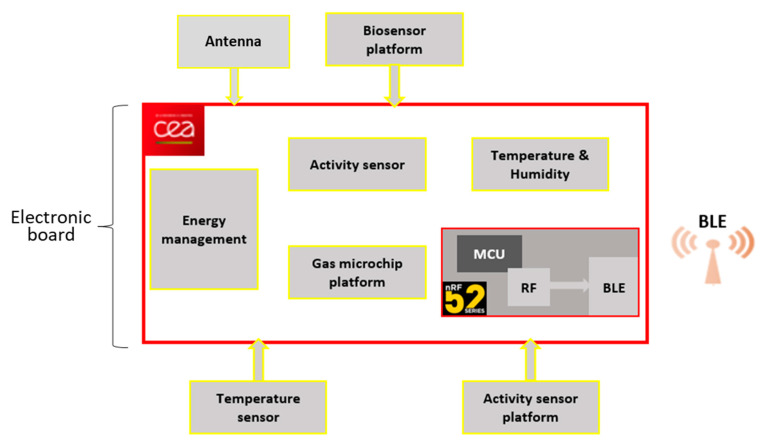
Low-power sensing platform simplified design. The red rectangle represents the electronic board integrating the MCU, the BLE, energy management and some sensors. Other sensors can be connected to the board.

The low-power sensing platform developed by CEA-LETI is described in Figure 1.

### 2.1. Electronic Architecture

This platform support both analog and digital sensors, developed by Convergence project partners, such as activity [4,11], sweat and pH [14] and gas sensors like NO_x_ [15,16], CO_x_ [7], NH_x_ compounds.

**Figure 2:**
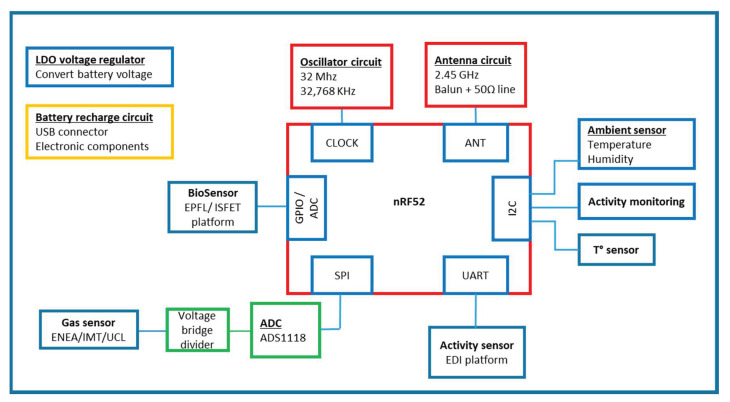
Electronic board (red rectangle) developed by CEA-LETI with communication protocols Serial Peripheral Interface (SPI), Universal Asynchronous Reception and Transmission (UART), Inter-integrated-circuit (I2C), General Purpose Input/Output (GPIO) converter, Analog to Digital Converter (ADC).

Gas (Convergence sensors), Capacitive Digital Humidity & Temperature (from STMicroelectronics, Crolles, France) and activity sensors (from Bosch, Reutlingen, Germany) are embedded on the flexible platform. Other sensors can be connected to the board, like the ISFET biosensor developed by EPFL (Lausanne, Switzerland), and the activity sensor developed by the EDI platform (Riga, Latvia). The architecture with the communication protocols is detailed in Figure 2 below.

In detail, the core of the electronic circuit was developed for sensor data acquisition and data transfer over Bluetooth Low Energy (BLE). The electronic architecture is based on a nRF52 BLE SoC from Nordic Semiconductor (Trondheim, Norway). The nRF52 SoC embeds an ARM-Cortex M4 processor with floating-point unit (FPU), 2.4 GHz transceiver, and contains 512 kB of flash memory and 64 kB of RAM that can be used for code and data storage. It offers optimized configuration, very low power performance and up to 32 available GPIOs. The communication protocols are specific to each sensor (UART, I2C, SPI) and converters are required for some sensors (GPIO/ADC). The sampling rate measurements of the different sensors are summarized in Table 1:

### 2.2. Antenna

#### 2.2.1. Antenna Specifications

The antenna circuit was designed by G-INP in order to work at 2.4 GHz [17,18] (bandwidth from 2.402 GHz to 2.480 GHz). The transmission power range is from −20 dBm to 4 dBm. The mandatory actual sensitive level for a Bluetooth receiver is −70 dBm or better. The typical sensitivity of Bluetooth receiver in mobile phone is −90 dBm. The expected communication distance is >10 m. Based on these conditions, Table 2 describes the calculation of desired antenna gain at different transmission powers. The antenna is expected to have a minimum gain of −9.77 dB.

The antenna was connected with the nRF52 chip via an adaptation circuit and input impedance of the antenna is 50 Ω. The antenna was then printed on the flexible substrate, covered by 6 mm of resin (flexible silicon) and protected by a thin fabric layer. The low-power sensing platform was then folded around the wrist like a smart watch. Table 3 shows the characteristics of the wrist tissue (four-layer model) of the human body.

The maximum realized gain of the antenna in air was 2.6 dB and the realized gain on the wrist was −5.73 dB. The estimated performance of maximum communication distance at (transmission power −20 dBm) is presented in Table 4.

#### 2.2.2. Antenna Simulated Results

**Figure 3:**
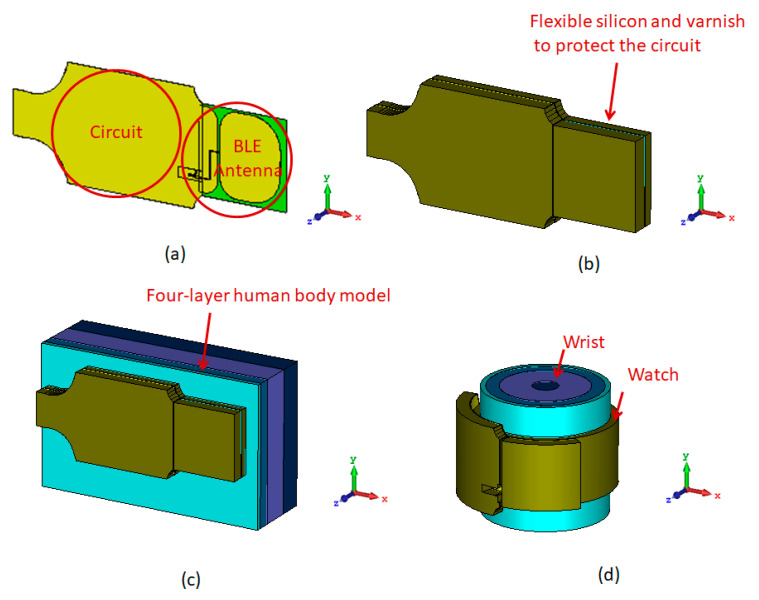
Configuration of antenna: (**a**) Integrated with the circuit (A1); (**b**) Protected by two layer of varnish and flexible silicon (A2); (**c**) Placed next to the human body (A3); (**d**) Bent around the human’s wrist (A4).

Simulations with CST MICROWAVE STUDIO were performed to optimize the antenna following different configurations represented in Figure 3:

- The antenna in air (A1)
- The antenna with protected varnish and resin in air (A2)
- The antenna with protected layers above human’s wrist (A3)
- The antenna with protected layers folded around human’s wrist (A4)

**Figure 4:**
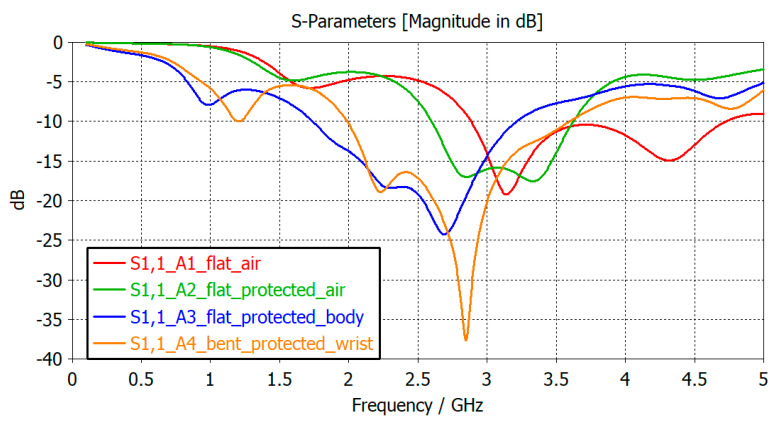
Simulated reflection coefficient of the proposed antenna in four scenarios: A1 placed in air; A2 protected by varnish and silicon; A3 placed on human body; A4 bent on human’s wrist.

Figure 4 presents the simulated results of reflection coefficient of proposed antennas at resonant frequencies in four cases.

**Figure 5:**
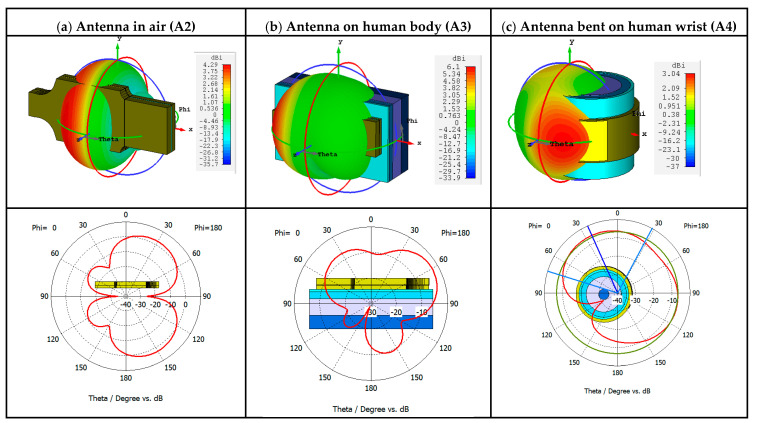
Radiation pattern of the antenna in 3D (directivity) and at φ =0 (realized gain): (**a**) A2 placed in air; (**b**) A3 placed on human body; (**c**) A4 bent on human’s wrist.

The performance of the antenna in the bending condition is important to evaluate the adaptability of the design in the wearable application. The radiation pattern of the proposed antenna at 2.45 GHz is presented in Figure 5.

The simulated gain and radiation efficiency are presented in Table 5.

### 2.3. Printed Circuit Board and Antenna Design

**Figure 6:**
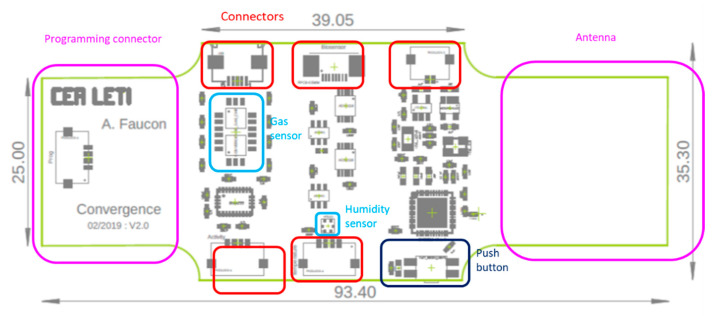
Flex PCB: design and dimensions.

The design of the flex Printed Circuit Board (PCB) is a rectangle of around 93.4 mm × 35.3 mm represented in Figure 6. The design was chosen to be worn as a wristband on the arm.

The flexible PCB materials specifications are described in Table 6. The substrate is kapton.

**Figure 7:**
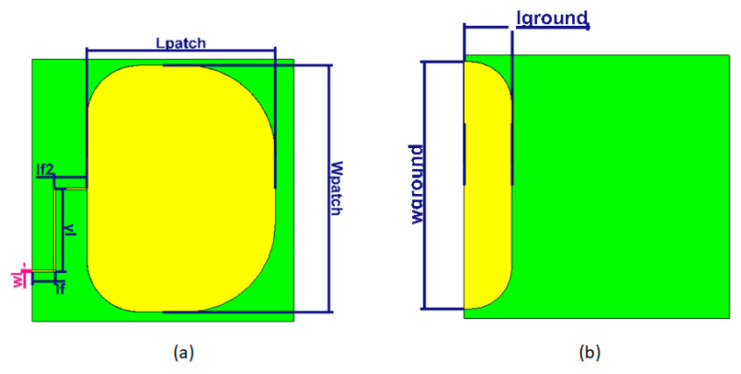
Configuration of the proposed antenna: (**a**) Top view; (**b**) Bottom view.

The antenna was integrated with the overall design as presented in Figure 3a. The antenna design used a transient solver within CST Microwave Studio for simulation, with permittivity of 3.4, tangent loss of 0.004 and thickness of 0.17 mm. The configuration of the proposed antenna is described in Figure 7.

The design values for all parameters are provided in Table 7. The dimension of the antenna is 24 × 23 mm^2^.

### 2.4. Consumption Test

The low-power sensing platform is a low-power system with different working modes. Different scenarios were tested to evaluate power consumption of the low-power sensing platform. The scenarios considered the embedded sensors: humidity, temperature and activity sensor. Results are summarized in Table 8.

**Figure 8:**
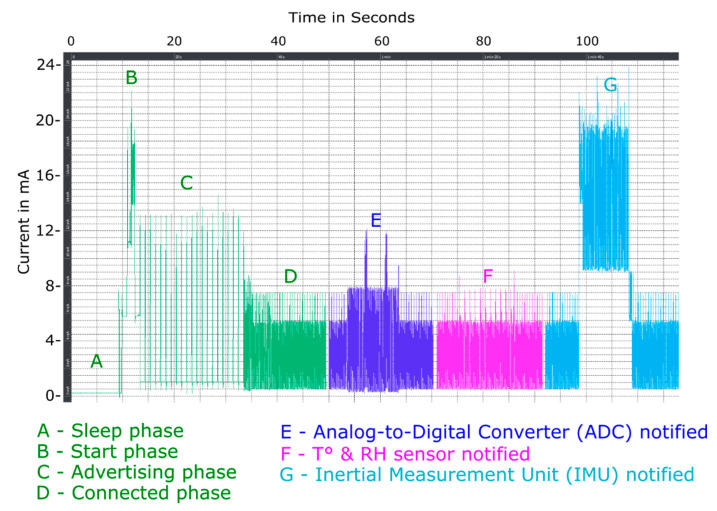
Power consumption, without external sensors, Sleep (**A**), Start (**B**), Advertising (**C**) and Connected Phase (**D**); Analog-to-Digital Converter notified (**E**); T° & RH sensor notified (**F**); Inertial Measurement Unit notified (**G**).

The different scenarios are represented in Figure 8.

The Inertial Measurement Unit (IMU) is the most energy-intensive component in comparison to other components embedded on the platform.

### 2.5. Application Development

**Figure 9:**
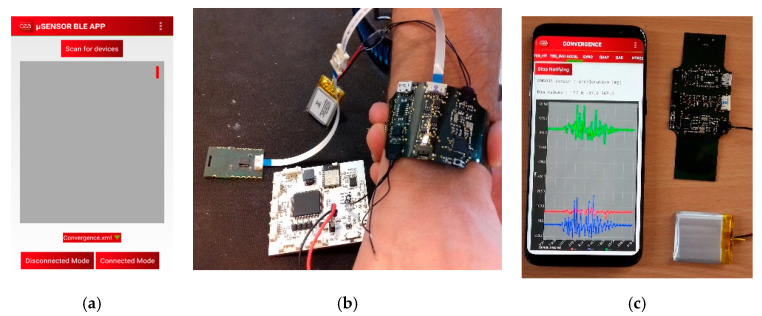
(**a**) Application interface (**b**) Flexible sensor platform tested with ISFET and activity sensor from EPFL and EDI—(**c**) Flexible sensor platform tested with embedded sensors.

CEA-LETI developed an Android application dedicated for the low-power sensing platform. The application allow the user to see in different windows the data of each sensor (Figure 9). The collected data are directly sent by Bluetooth Low Energy on a smartphone in order to allow their visualization in real-time.

### 2.6. Integration

**Figure 10:**
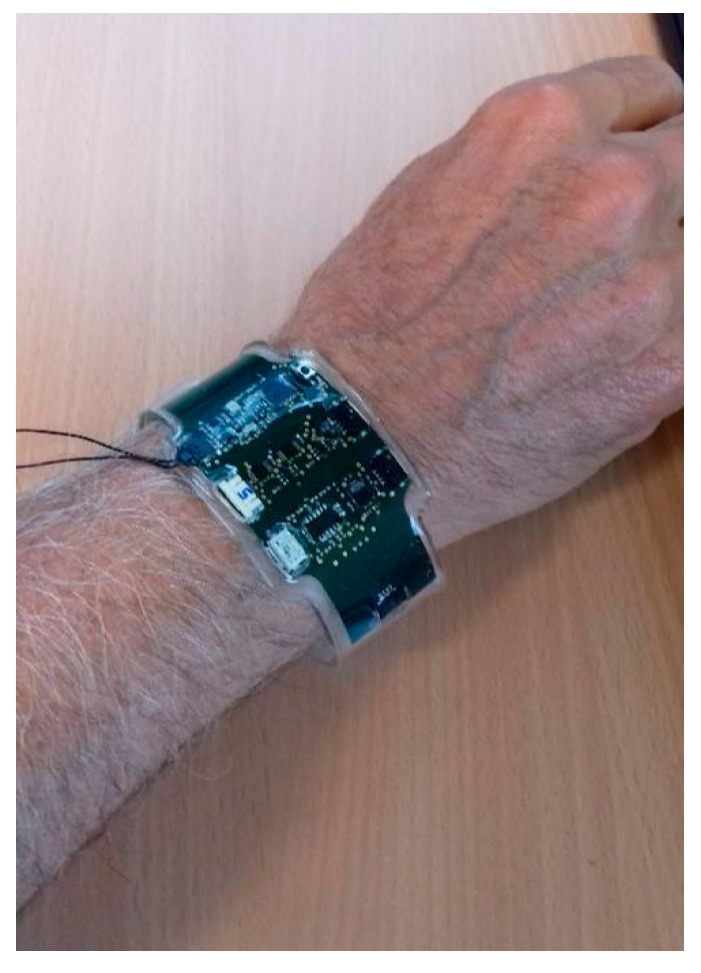
Low-power sensing platform integration in Sylgard^®^ 184 silicon.

The flex PCB was encapsulated into Sylgard^®^ 184 silicon using a mould with a wristbands shape (Figure 10). The mould was manufactured using a 3D printer and machined in order to avoid roughness. Indeed, low roughness was needed to ensure a good transparency of the device. A first layer of Sylgard^®^ 184 was deposited in the low part of the mould and cured at 80 °C during 10 h. After that, the flex PCB was placed into the mould and a second layer of Sylgard^®^ 184 was added to recover it. A second curing at 80 °C during 10 h was then performed. Some integration constraint were taken into account for the integration like the shape, the size and the conformability of the low-power sensing platform. For the good operation of the platform, some “open windows” for the sensors and the connectors were done and represented in Figure 6. These open windows were implemented by adding some putty in the connexion zones and in the gas and humidity sensors zones. The putty was removed after the second curing.

## 3. Sensors Characteristics

The smart sensing platform is compatible with different sensors developed in the Convergence project, like for example:

- A bio-sensor, an ISFET sweat/pH sensor developed by EPFL [14]. The working principle similar to a MOSFET.
- Gas sensors: a miniaturized gas sensor combining NO_2_, CO and NH_3_ gases on the same dye; with NO_2_ sensor developed by ENEA, NH_3_ sensor by UCL and CO sensor by IMT [7].
- Humidity and Temperature sensors from STMicroelectronics, which are very low power with approximately 2 µA consumption @ 1 Hz output data rate. It is connected to µC via I2C bus and may be powered from 1.7 V to 3.6 V.
- Activity sensor developed by EDI [4].

Here, we report the current results of development activities concerning the NO_2_ sensor based on graphene and CO sensor. These sensors are two of the three gas sensors developed in view of a miniaturized dye.

The micro dye hosting several sensors idea takes inspiration from electronic nose concept. The electronic nose uses an array of transducers, each one deposited with a different material and responding preferentially to a certain gas. In the present work, we are using two versions of dies with 2 × 2 and 3 × 3 miniaturized interdigitated electrodes (IDEs). On a 2 × 2 die, the IDEs area is equal to 1 × 1 mm^2^, with digit/gap dimensions of 4/4 µm. On a 3 × 3 die, the area delimited by the IDEs is equal to 200 × 300 µm^2^ and the digit/gap dimensions are 2.5/2.5 µm. On each die, we deposited three different sensitive materials according to the goals, for detection of NO_2_, CO and NH_3_.

The miniaturization phase is ongoing. The three sensors on a die will be tested in each of the three gases and combination of these gases and the calibration curves will be obtained. To overcome the cross-sensitivity issues suitable processing algorithms will be applied to the signals.

To implement the concept of three sensors in a die we agreed to develop InkJet printable materials which was the most appropriate method to selectively deposit the sensitive material directly on the active area of the IDEs. The ink-jet printing method offers the advantage of precise deposition on small surfaces, and the possibility to adjust the printing parameters such as droplet volume, droplet distance, and the number of layers. For each sensor, a different material was developed taking into consideration the affinity (documented in previous work) [7,16] of the material with the targeted gas.

In the following part, the development of NO_2_ sensor based on graphene and on CO sensor development are described.

### 3.1. NO_2_ Sensor: Synthesis of the Sensing Materials

In a wearable device, the use of materials able to work at low temperatures with very low dissipated power is crucial. Graphene-based materials are the perfect candidates for this purpose, being highly sensitive and selective towards NO_2_ at room temperature. In the following sections, the preparatory tests for the integration of graphene-based sensing devices into the low-power sensing platform are to be described.

According to our experience bare graphene (Gr) and graphene functionalized with ZnO (Gr-ZnO) were selected as sensing layers. Pristine graphene was synthetized by exfoliating graphite flakes in a hydro-alcoholic solution by a sonication-assisted method [19]. In detail, graphite flakes were dispersed into a mixture of a water/IPA mixture (7:1 *v*/*v*) and the dispersion was sonicated in a low power bath for 48 h. The subsequent centrifugation at 500 rpm for 45 min allowed separating from the dispersion unexfoliated graphite crystallites, thus obtaining a black suspension of few-layer graphene with a final concentration of 0.1 mg mL^−1^.

In order to produce the graphene nanocomposite with ZnO, 20 mL of graphene dispersion were added with ZnO nanoparticles (∅ 14 nm), in stoichiometric molar ratio C:Zn of 3:1, sonicated briefly to disperse the ZnO particles and freeze-dried so to attain a dry mix of graphene and ZnO. The powder was then microwave irradiated at 1 kW for 5 min. The final ZnO-graphene nanocomposite powder was finally dispersed in ethanol [16].

### 3.2. NO_2_ Sensor: Materials Characterizations

Both bare Gr and Gr-ZnO nanocomposite were structurally and morphologically characterized through several techniques: Field-Emission Scanning Electron Microscopy (Zeiss-LEO 1530-2 FESEM microscope, Oberkochen, Germany) with an acceleration voltage 5 kV was employed to assess the morphology and the composition of the investigated samples. Structural data were acquired by means of Raman spectroscopy performed through an InViaReflex spectrometer (Renishaw, Wotton-under-Edge, UK) for 514 nm wavelength incident laser light, in backscattering configuration. Additional information about the samples structure and morphology were achieved by the transmission electron microscopy (TEM TECNAI G12 Spirit-Twin, FEI/Thermo Fisher scientific, Waltham, MA, USA). I-V measurements were recorded on devices fabricated using Gr-ZnO and pristine graphene in the range between −10 V and 10 V through a semi-automatic probe-station (2636A Dual-channel System Source Meter, Keithley, Cleveland, OH, USA).

### 3.3. NO_2_ Sensor: Device Fabrication and Gas Sensing Protocol

For the preliminary sensing tests described herein, simple laboratory-testing chemiresistors were fabricated. Gr and Gr-ZnO suspensions were casted onto rough alumina substrate with pre-printed Au interdigitated electrodes (350 μm wide, 4650 μm long and spaced of 350 μm) which delimit a sensing area of around 0.5 mm^2^.

The sensing measurements were carried out in an airtight chamber (40 cl) with electrical feed-through; the devices were biased at 1 V by means of a TTi QL355T Precision Power Supply and the conductance values was recorded by a high-resolution Keithley 6485 picoammeter. A water bubbler placed in a thermostatic bath humidifies the dry air carrier gas and allows adjusting the relative humidity to a pre-defined value. During the measurements the total flow is kept at 500 sccm using N_2_ as carrier gas, the temperature at 22 ± 2 °C and at ambient pressure. The measurement protocol is composed of 3 phases: (1) 20 min of device exposure to carrier gas (baseline); (2) 10 min of exposure to the target gas (response to the analyte); (3) 20 min of exposure in carrier gas to restore the initial conductance value of the device (recovery).

The response *R* of the gas device is defined as:

$$R\left(\%\right)=\frac{\left(G_{max}-G_{0}\right)}{G_{0}}\times 100$$

where *G_max_* is the conductance value reached at the end of the exposure window and *G_0_* is the conductance value before exposure.

### 3.4. CO Sensor: Preparation of Inkjet Material and Deposition

Materials used for the synthesis are ammonium persulfate (APS), aniline, SWCNT-COOH, Tween 80, lactic acid, poly(styrene sulfonate) (PSS), phosphate buffered saline (PBS) (pH 7.4), and acid acetic purchased from Sigma (Redox, Romania). The formula ink-jet preparation is the following:

S1 solution: 0.3 g PSS, 3.2% SWCNT in 1 M acetic acid; ultrasonication for 3 h at room temperature. Then 25 µL aniline was added dropwise.

S2 solution: 0.075 g APS adding to S1 solution and stored for 24 h in dark conditions.

The final PANI:PSS/SWCNT inkjet formula solution is a mixture of solution S2 with PBS 2:1 (*v*/*v*), 2% lactic acid and 1% Tween 80, ethylene glycol (viscosity = 12 cP).

Method: A DIMATIX DMP 2800 printer (FujiFilm Dimatix, Lebanon, NH, USA) has been used to print the sensor active area with PANI:PSS/SWCNT ink. The cartridge is filled with 1.5 mL of freshly synthesized PANI-PSS/CH_3_COOH ink and is loaded into the printer. Printing tests are performed to check the fluidity and dispersion of the ink on the substrate. The following step consists in setting the printing parameters: optimal operating pulse 9344 µs, pulse frequency: 5 kHz; temperature: 30 °C; piezoelectric nozzle voltage: 28 V; drop spacing: 20 µm, number of printing cycles: 5.

The first step in the printing process is to create the desired layout using integrated CAD software. The maximum printing substrate is 22 cm × 30 cm. After placing and fixing the substrate, printing starting point (print origin) and the number of printing cycles, the printing process can start. If there are several wafers placed on the plate, printing can be done on all wafers at the same time, with geometric configuration settings being made in advance. If more than one printing layer is required (in our case), the process is repeated automatically, no further parameter adjustments needed during the printing process.

## 4. Tests and Results

### 4.1. NO_2_ Sensor: Results and Discussion

**Figure 11:**
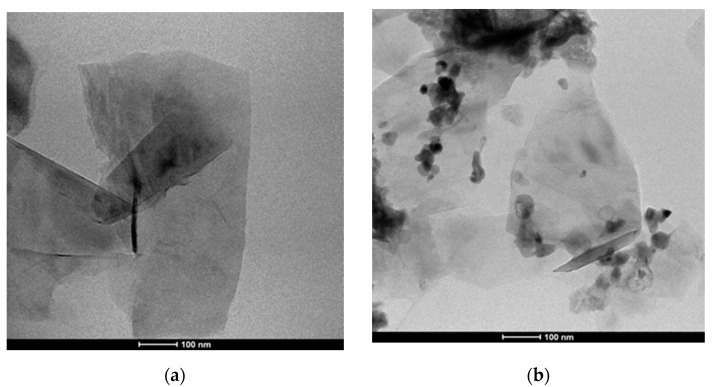
TEM photographs: (**a**) pristine graphene flakes with lateral size of several hundred nm folded and casually overlapped; (**b**) Gr-ZnO/NP nanocomposites. The nanoparticles are mainly amassed along the edges of the flakes.

**Figure 12:**
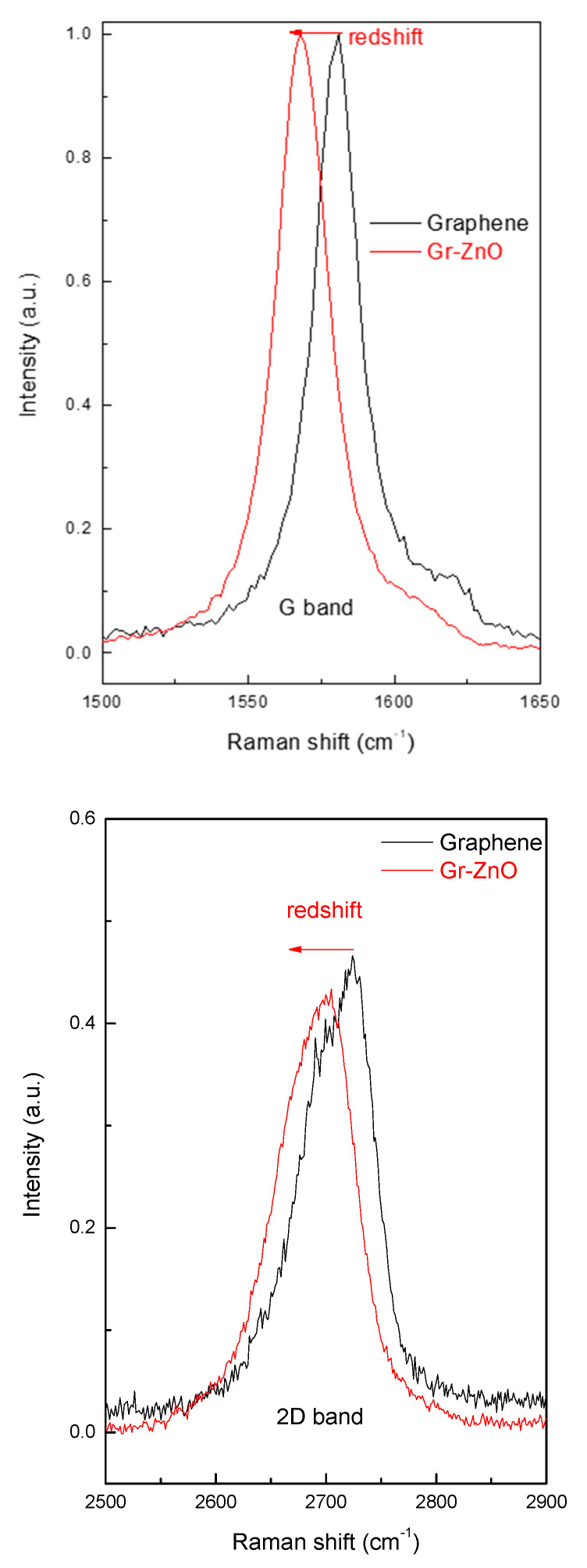
The shift of G and 2D bands in the Raman spectrum of graphene functionalized with ZnO nanoparticles in comparison with that of pristine graphene.

**Figure 13:**
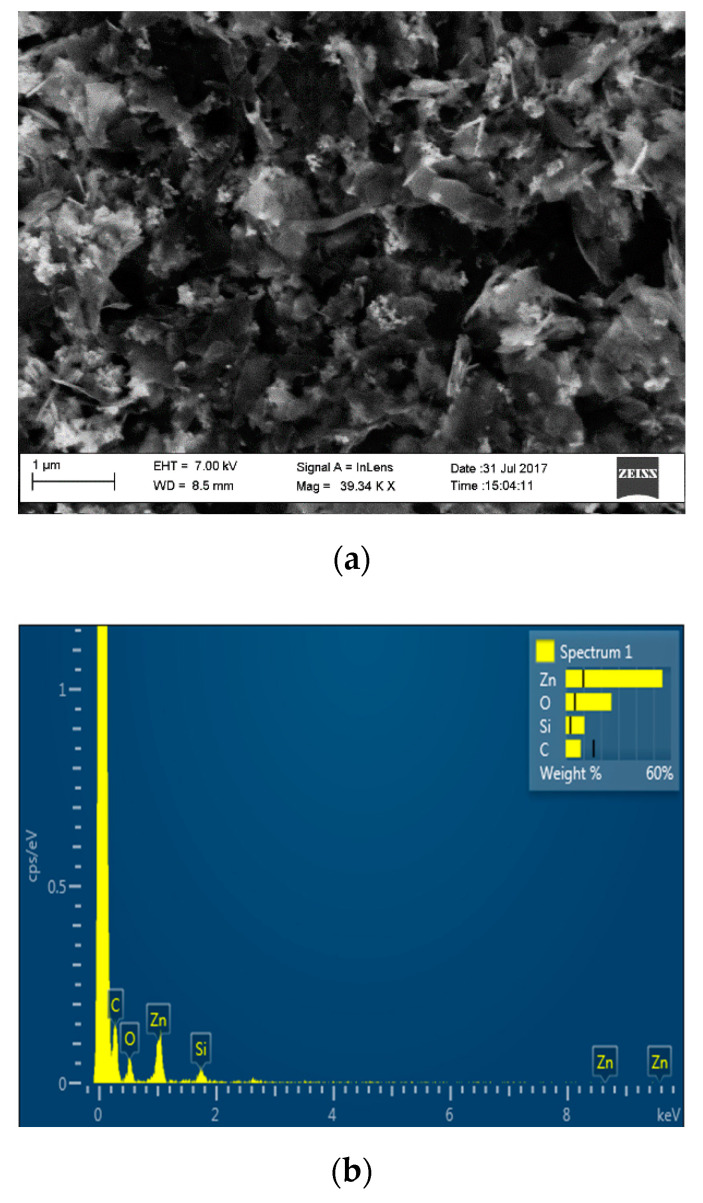
(**a**) Gr-ZnONPs nanocomposites observed under SEM and (**b**) EDX spectrum collected in an area surrounding ZnO nanoparticles.

In the following Figure 11, Figure 12 and Figure 13, the whole sequence of morpho-structural characterizations of the prepared materials, consisting of transmission electron microscopy (TEM) imaging, Raman spectroscopy, scanning electron microscopy (SEM) and energy-dispersive X-ray spectroscopy (EDX) analysis is portrayed.

The characterizations on the pristine material indicate that it is composed of a few layers of graphene: the TEM image (Figure 11a) shows thin graphene flakes with a lateral dimension of a few hundred nm. At the same time, the shape of the 2D peak in the Raman spectrum of Figure 12 proves the effective material exfoliation down to five layers, as discussed in more detail in [17]. As for the hybrid material, the TEM image (Figure 11b) shows graphene flakes coated with nanoparticles and distributed mainly on the edges rather than in the basal plane. A wide view of the hybrid morphology is presented in the SEM image (Figure 13a), that basically confirms the nanostructure highlighted by TEM imaging. The EDS analysis provides the composition of the material in the surrounding of the nanoparticles showing four dominant peaks: Zn, S, C and O, confirming that nanoparticles are composed of ZnO. The shift of the G and 2D peaks of the Raman spectrum of the hybrid material with respect to those of bare graphene, suggests a variation in the doping level of the material and therefore a strong interaction between nanoparticles and graphene.

For the device characterization, first, the ohmicity of the contact between Au and the graphene-based materials was verified with volt-amperometric measurements and the value of basic resistances are summarized in Table 10.

The devices were tested towards low concentration of NO_2_ (0.1–1.2 ppm) diluted in humidified N_2_ at room temperature.

**Figure 14:**
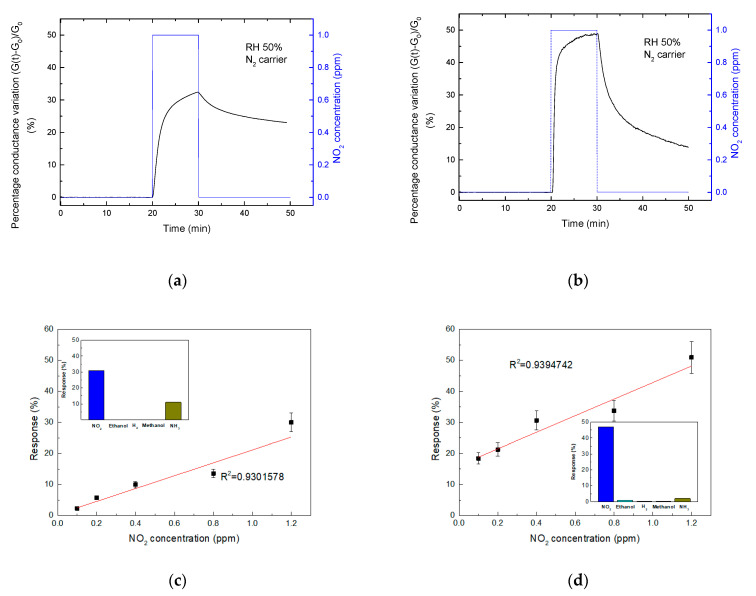
Sensing responses to a single pulse of 1000 ppb NO_2_ of chemiresistors based on graphene (**a**) and ZnO decorated graphene (**b**). Sensing response of the graphene-based device (**c**) and of the device based on the ZnO functionalized graphene (**d**) in the range 0.1–1.2 ppm NO_2_; the linear regression is reported as red line along with the regression coefficient. In the insets the selectivity towards 1 ppm NO_2_, 50 ppm ethanol, 1% hydrogen (H_2_), 50 ppm methanol and 250 ppm ammonia (NH_3_) of both materials.

Figure 14a,b show typical responses of the pristine graphene and Gr-ZnO-based sensors vs. 1 ppm NO_2_; the related tests for different NO_2_ concentration in 0.1–1.2 ppm range are reported in Figure 14b,c, respectively. The regression lines (in red) with the respective coefficients are also reported, showing a nearly linear trend. Both devices were also exposed to several analytes, such as NH_3_, hydrogen, ethanol and methanol; in all cases they exhibited a significantly lower response than that to NO_2_ (see the insets in Figure 14b,c, respectively), thus demonstrating their specificity towards this analyte.

### 4.2. CO Sensor: RESULTS and Discussion

Starting from Chiolerio’s study [20] that showed that PANI can be used for the synthesis of functional inks for inkJet printing of thin polymeric layers, we developed a dedicated ink as a sensitive layer for CO detection to be deposited on the sensor electrodes. For this purpose, an ink formula with conductive particles consisting of polyaniline, polystyrene sulfonate and carbon nanotubes (PANI:PSS/SWCNT) has been synthesized. The PANI/SWCNT (polyaniline/single-wall carbon nanotube) ink material has been developed to be printed through ink jet process on the CO sensor active area. The main requirements which have driven the development of the inkjet printing solution are: viscosity of 10–12 cP, surface tension of 28–33 dyn/cm, boiling point >100 °C, 4 < pH < 9. Moreover, for the inkjet solution to be used in sensor applications, the electrical conductivity needed to be customized to fulfill the application needs.

Table 9 shows the PANI:PSS/SWCNT ink jet formulation after synthesis in comparison with PANI ink jet solution. The green color of the PANI solution is due to emeraldine salt form and indicates that the conductive PANI form has been obtained. After adding the SWCNT, the formulation became dark green.

**Figure 15:**
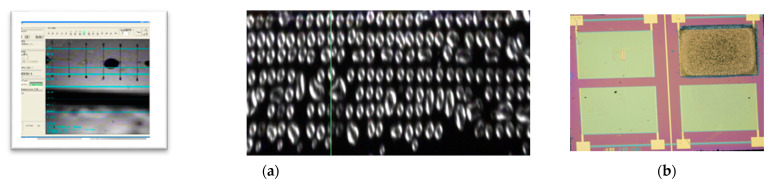
(**a**) Images during the printing process of the PANI:PSS/SWCNT ink with Dimatix DMP 2800; (**b**) Optical microscope image if PANI:PSS/SWCNT on one pair of IDE.

The printing results are shown in Figure 15. Figure 15a represents the camera image of the IDEs surface during printing with the Dimatix DMP 2800 system Figure 15b shows the visible spots on the optical microscope of the sensor surface after IDEs printed with ink-jet formulation. The solution shows a good viscosity for printing process and therefore after drying shows a uniform deposition on IDEs (X.B).

**Figure 16:**
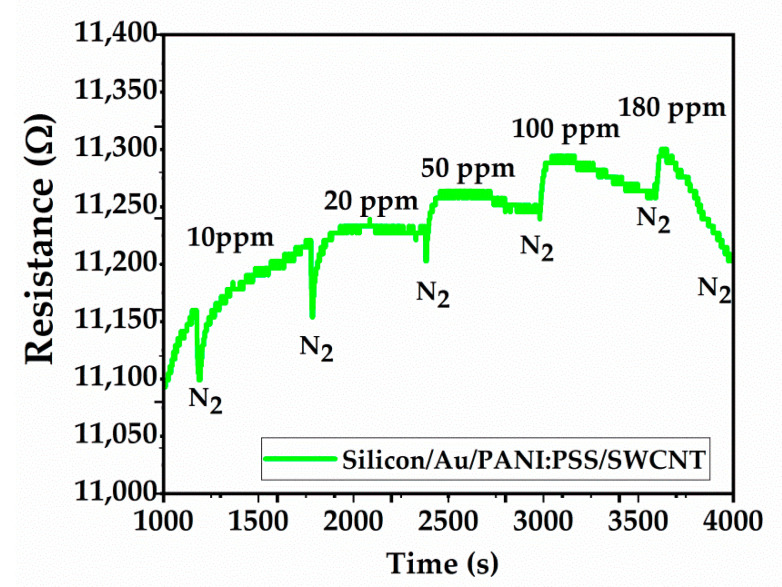
Sensor resistance (in Ω) versus time (in seconds) under CO exposure.

The exposure of the PANI:PSS/SWCNT sensor to CO gas is presented in Figure 16. The increase of the sensor resistance in response to CO concentration is observed. The sensor was exposed to CO for 10 min and the recovery time was 20 min in N_2._

**Figure 17:**
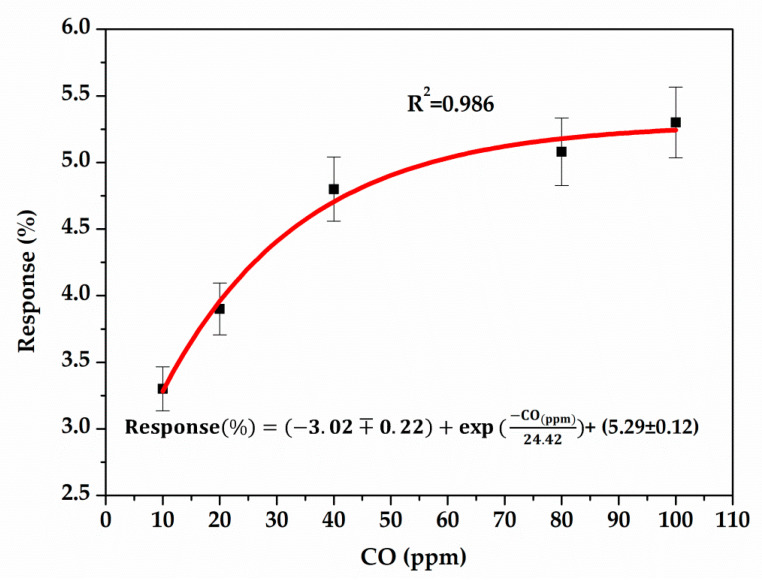
Sensor response versus CO concentration.

Figure 17 shows the sensitivity of the PANI:PSS/SWCNT layer using the Equation (1) in the presence of five different CO concentrations. The sensor’s response increases with the increase of CO concentration:

(1) $$S\left(\%\right)=\frac{\left|R_{g}-R_{a}\right|\;}{R_{a}}\times 100$$

where *Rg* is the resistance of the sensor after each added CO concentration and *Ra* is the resistance in air.

### 4.3. NO_2_ Sensor Tests with Low-Power Sensing Platform

In Table 10, we report the main features of the laboratory-testing chemiresistors based on graphene.

**Figure 18:**
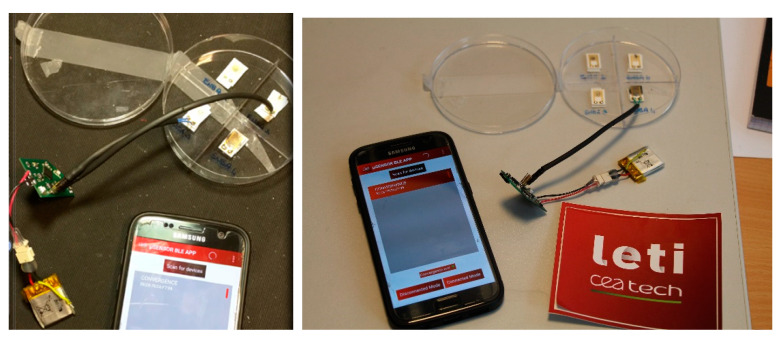
The graphene-based NO_2_ sensors connected to the CEA-LETI platform.

As shown in Figure 18, the sensors were plugged onto the sensor platform that converts the analog electric signal into a measurable signal through an ADC Voltage Divider Bridge.

Table 11 displays the main electrical parameters measured after the connection to the platform. The resistance values, both measured by a multimeter and by the platform, are in agreement with an error of about 1%, thus indicating that the connection to the platform did not introduce any contact resistance.

**Figure 19:**
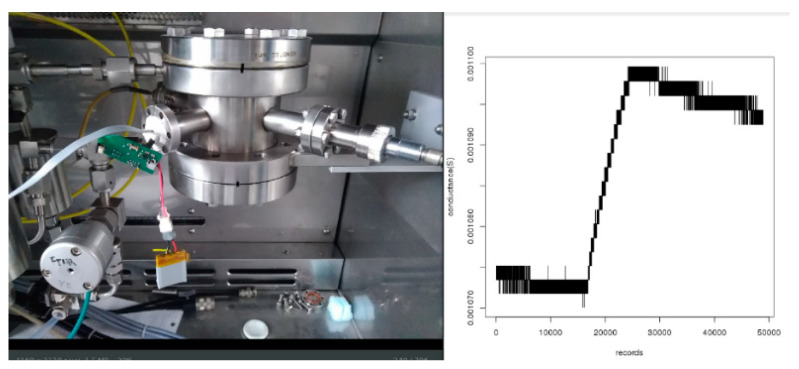
Pristine graphene-based sensor, named ENEA3, installed on LETI board, exposed to 300 ppb of NO_2_ for 10 min.

After these characterizations, devices ENEA 3 and ENEA 4, connected to the CEA platform, were tested into ENEA sensor test chamber with the measurement protocol described above. As can be seen in Figure 19. ENEA3 device installed on LETI board and exposed to 300 ppb NO_2_, exhibits a variation of 3%, thus demonstrating that the platform is able to follow and display in real time on a smartphone the signal variation consequent to the exposure to such analyte.

**Figure 20:**
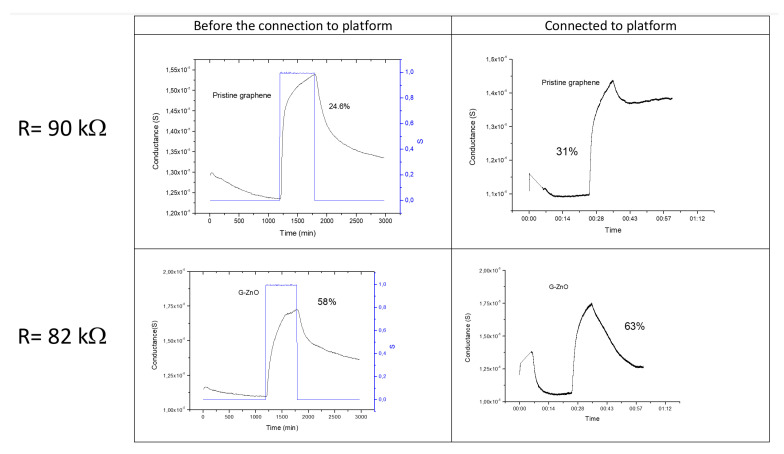
Sensing responses of the newly prepared devices, based on pristine and decorated graphene, exposed to 1 ppm of NO_2_ for 10 min. Figure is cut off.

However, a certain mismatch among the technical features of the sensors before and after connection can be observed, which was probably due to an incorrect analog-to-digital conversion. Indeed, by adjusting the resistance values of the sensor devices as close as possible to that of the Voltage Divider Bridge (67 kΩ) an optimal signal digitalization was achieved. According to this, new devices, based on pristine and decorated with ZnO nanoparticles graphene have been prepared: the base resistances, measured by a multimeter before the connection to the platform, were 90 kΩ and 82 kΩ for pristine and decorated graphene, respectively. In Figure 20, the results of the exposure towards 1 ppm NO_2_ of the devices in both the configuration, connected or not to the platform, are depicted.

The comparison between the device performances, before and after the connection to the platform, was carried out by evaluating the base resistance, averaged over 20 min in inert environment, and the conductance variation caused by the exposure to the analyte and summarized in Table 12:

As for the base resistance, the obtained values are consistent within 8–14%, but it is worth noting that their discrepancy is mainly attributable to the incomplete sensor restoration conditions.

With respect to the sensing response, again in this case the conductance variations are affected by their initial value, so that in each device the most consistent variation is observed when the conductance starts from a lower value. In any case, the conductance variations before and after the connection to the platform are in agreement within 8–25%.

## 5. Conclusions

In this paper, a low-power sensing platform for health and environment monitoring with embedded sensors and external sensors was developed and tested. The resulting integrated device results from research activities carried out by several partners in the framework of the Convergence project.

The platform is a low power system working with BLE and compatible with different kind of sensors to monitor not only the health of individual person (physical activity, core body temperature and biomarkers) but also the environment with chemical composition of the ambient air (NOx, COx, NHx particles). A specific antenna was designed for our specific application with expected communication distance above 10 m. Low-power sensing platform was integrated in silicon in order to be wear as a bracelet.

For the integration of the gas sensors into the platform, the focus was on devices operating at room temperature such as NO_2_ sensor by ENEA, and CO sensor by IMT.

Graphene-based sensors, able to detect few ppb NO_2_, were suitably designed and plugged onto the CEA-LETI board; the ability of the platform to correctly reproduce the detection response of the graphene-based device under controlled conditions in a gas chamber was therefore demonstrated.

Besides, a CO sensor was developed using a PANI/SWCNT sensitive layer in the form of a printable ink, deposited with a DimatixDMP2800 printer and polymerized at 60 °C for 2 h. The CO sensor functionality was demonstrated.

The illustrated work is preparatory to the realization of sensor micro-arrays, which will allow the actual finalization of the wearable device.

## Figures and Tables

**Table 1:** Sampling rate of the different sensors.

Sensors	Sampling Rate (Hz)
Embedded activity sensor	10
Embedded Temperature & humidity sensor	1
Gas	10
Activity platform (EDI)	2
ISFET sweat/pH biosensor	1
Temperature	2

**Table 2:** Calculation of desired antenna gain for different scenarios.

Conditions	Communication Distance	Transmission Power	Receiver’s Sensitivity	Desired Antenna Gain
Worst scenario	10 m	−20 dBm	−90 dBm	−9.77 dB
Best scenario	10 m	4 dBm	−90 dBm	−33.77 dB

**Table 3:** Characteristic of four-layer model human’s wrist.

Tissue	Radius (mm)	Permittivity	Loss Tangent
Skin	2	38.06	0.28
Fat	5	5.29	0.15
Muscle	12	52.79	0.224
Bone	10	18.49	0.25

**Table 4:** Estimated maximum distance of the complete system.

Conditions	Transmission Power	Receiver’s Sensitivity	Antenna Gain	Maximum Distance
In air	−20 dBm	−90 dBm	2.65 dBi	41.5 m
On wrist	−20 dBm	−90 dBm	−2.44 dBi	23.0 m
On wrist (folded)	−20 dBm	−90 dBm	−5.73 dBi	15.8 m

**Table 5:** Simulated performance of the proposed antenna.

Antenna (with Protected Resin) at 2.45 GHz	Reflection Coefficient (dB)	Realized Gain (dB)	Total Efficiency (%)
Antenna in air (A2)	−6.5	2.65	68.6%
Antenna on wrist (A3)	−18.5	−2.44	13.9%
Bended antenna on wrist (A4)	−16.5	−5.73	12.1%

**Table 6:** Materials specifications.

	Material	Thickness	Characteristics
**Substrate**	Kapton	0.05 mm	Relative Permittivity: 3.3 Tan (δ): 0.004 @ 2.45 GHz
**Conductor**	Copper	0.0035 mm	Conductivity: 5.8 × 10^7^ S/m
**Protect**	Varnish	0.0025 mm	Relative Permittivity: 4.3 Tan(δ): 0.03
**Resin**	Flexible Silicon	3 mm below circuit 5 mm above circuit	Relative Permittivity: 2.8 Tan(δ): 0.0015 @ 1 MHz

**Table 7:** Optimized parameter values of the proposed antenna.

Parameter	Value (mm)	Parameter	Value (mm)	Parameter	Value (mm)
Wpatch	24	lf	3	wground	24
Lpatch	18	lf2	3	lground	4.5
wl	0.15	yl	8		

**Table 8:** Power consumption of embedded sensors on the test platform depending on the scenario: OFF or Low Power (LP) mode. **Why does text in the columns not wrap?**

	Scenario	Consumption
**Static mode** (A)	nRF52 configuration: **OFF Mode** BLE communication disabled All peripherals/GPIOs disabled	760 µWh P_avg_ = 0.76 mW P_max_= 0.76 mW
**Dynamic mode** (B,C)	nRF52 configuration: **LP mode** BLE communication enabled Sending connection request (**advertising packets every 1 s**) Sleep mode for internal sensors	3.9 mWh P_max_ = 52 mW
**Dynamic mode** (D)	nRF52 configuration: **LP mode** BLE communication enabled Mode connected + notifications enabled Waiting sensor notification (**L2CAP packets every 100 msec**) Sleep mode for internal sensors	4.1 mWh P_max_ = 29 mW
**Dynamic mode** (G)	nRF52 configuration: **LP mode** Mode connected + notifications enabled **Inertial Measurement Unit (IMU): acquisition measures** **(accelerometer, gyrometer & quaternion) + sending data** **(20 bytes) at 10 Hz** T&RH sensor: sleep mode Analog-to-Digital Converter: sleep mode	42.8 mWh P_avg_ = 43 mW P_max_ = 78 mW
**Dynamic mode** (F)	nRF52 configuration: **LP mode** Mode connected + notifications enabled Accelerometer: sleep mode **T&RH sensor: acquisition + sending data (4 bytes) at 1 Hz** Analog-to-Digital Converter: sleep mode	4.2 mWh P_avg_ = 4.2 mW P_max_ = 29 mW
**Dynamic mode** (E)	nRF52 configuration: **LP mode** Mode connected + notifications enabled Accelerometer: sleep mode T&RH sensor: sleep mode **Analog-to-Digital Converter: acquisition + sending data** **(16 bytes) at 10 Hz**	6.7 mWh P_avg_ = 6.7 mW P_max_ = 45 mW

**Table 9:** Ink Jet solutions properties.

Name of Ink-Jet Formulation	Conductivity (mS·cm^−1^)	pH	Viscosity (CP)
PANI: PSS (EG/Tween 80%)	2	4.0	8
PANI:PSS/SWCNT (PSS:Lacticacid:EG; Tween 80%)	4.98	6.0	12

**Table 10:** Summary of the basic resistance values of the sensors produced by ENEA and the corresponding sensitivity towards NO_2_.

	Sample Name	R (kΩ)	Sensitivity to NO_2_
Pristine graphene	ENEA 1	0.46	37% @ 300 ppb
Pristine graphene	ENEA 2	0.4	31% @ 1 ppm
Pristine graphene	ENEA 3	1.9	23% @ 1 ppm
ZnO NP decorated graphene	ENEA 4	88	50% @ 1 ppm

**Table 11:** Summary of the main electrical parameter checked after the connection to the CEA-LETI platform.

	Multimeter Measures [Ω]	Platform Measures [Ω]	Error [%]	Value Converted by the ADC [V]
ENEA2	593	598	0.8%	145
ENEA3	1984	2009	1.26%	477

**Table 12:** Summary of the main electrical parameter checked after the connection to the CEA-LETI platform.

	Multimeter Measurements [kΩ]	Base Resistance Not Connected to Platform [kΩ]	Base Resistance Connected to Platform [kΩ]	Conductance Variation Not Connected	Conductance Variation Connected
Pristine graphene	90	99	91	25%	31%
G-ZnO	82	79	90	58%	63%

## Data Availability

Not applicable.
